# Improving Urology Ward Round Documentation Through Sequential Digital Interventions: A Three-Cycle Quality Improvement Project

**DOI:** 10.7759/cureus.117436

**Published:** 2026-10-04

**Authors:** Sohaib Ansari, Samarth Golash, Marina Leonidou, Eman Ali, Eshan Shah

**Affiliations:** 1 Urology, University Hospitals of North Midlands NHS Trust, Stoke-on-Trent, GBR

**Keywords:** clinical audit, documentation standards, quality improvement, urology, ward round documentation, ward round proforma

## Abstract

Introduction

Complete ward round documentation is essential for safe and effective clinical decision-making, continuity of care, and multidisciplinary communication. Concerns around the completeness and accessibility of handwritten ward round entries in a urology department of a tertiary centre prompted a clinical audit, which identified omissions in notes. This quality improvement project evaluated changes in ward round documentation completeness across sequential audit periods following the introduction of electronic free-text documentation and subsequently a structured electronic proforma, using standards adapted from the Royal College of Surgeons of Edinburgh SHINE Surgical Ward Round Toolkit.

Methods

The project consisted of successive audit and intervention cycles. Ward round entries were assessed for 11 documentation criteria and coded as documented, not documented, or not applicable. Baseline data were collected from handwritten documentation from December 2024 to April 2025 (n = 32). Electronic free-text ward round documentation was then implemented, and these notes were re-audited from January 2026 to May 2026 (n = 43). Following this, an electronic proforma was launched, and a third audit of notes in the post-proforma implementation period was completed from June 2026 to July 2026 (n = 50), assessing both proforma and continued free-text ward round entries. The primary outcome was per-entry completeness, calculated as documented applicable criteria divided by all applicable criteria. Aggregate compliance was reported descriptively.

Results

Documentation completeness was progressively higher across the three sequential audit periods. Median per-entry completeness increased from 27.3% with paper documentation, to 50% with electronic free-text documentation, and to 60% in the post-proforma implementation period. Aggregate compliance increased from 29.2% to 48.3% and 59.3%, respectively. The proforma was used in 26 out of 50 (52%) post-implementation entries. Proforma entries had a higher median completeness than contemporaneous free-text notes (63.6% vs. 55.1%).

Conclusions

Structured ward round templates and interventions are known to improve the quality of ward round documentation. This quality improvement project saw ward round documentation completeness increase across sequential audit periods. Future work should address barriers to proforma adoption and target persistently under-documented criteria.

## Introduction

Ward rounds are a central mechanism for systematic inpatient review, multidisciplinary decision-making, and care planning [1]. Ward round notes act as a shared account of the patient’s current condition, investigations, treatment, and escalation plan. Incomplete or poor-quality documentation may impair handover and continuity of care, particularly in surgical environments where multiple teams contribute to care, with potential implications for patient safety [2,3].

Existing reviews suggest that checklists, templates, and structured ward round interventions can improve documentation quality and may support inpatient care and safety [4,5]. Individual quality improvement studies have reported improved documentation after implementation of ward round templates or checklists [6-8].

The Royal College of Surgeons of Edinburgh (RCSEd) SHINE Surgical Ward Round Toolkit provides a practical framework for safer and more consistent surgical ward rounds [9]. Within the urology department of a tertiary centre in the United Kingdom, ward rounds were documented on handwritten paper notes despite the existing availability of iPortal, an electronic clinical record system [10]. Anecdotal concerns around the accessibility and quality of these notes prompted a baseline audit, which showed significant omissions of patient-related information. In this quality improvement project, we therefore undertook steps to transition to electronic free-text documentation and ultimately to introduce a stakeholder-informed structured electronic proforma adapted from the RCSEd SHINE Surgical Ward Round Toolkit.

The primary aim was to evaluate changes in per-entry documentation completeness, assessed against criteria adapted from the RCSEd SHINE Surgical Ward Round Toolkit, across three audit periods, including paper documentation, electronic free-text documentation, and the post-proforma implementation period, which included both ward round entries utilising the proforma and those continuing to use electronic free-text. Secondary aims were to compare per-entry completeness between proforma and contemporaneous electronic free-text entries within the post-proforma period, measure real-world proforma uptake, and identify criteria requiring further improvement. The project evaluated documentation process measures and did not assess clinical outcomes.

## Materials and methods

Setting, context, and ethics

This quality improvement project was undertaken in the inpatient urology department of a tertiary care centre (Royal Stoke University Hospital, Stoke-on-Trent, United Kingdom). It consisted of two iterative interventions and three audit periods: a baseline audit and two re-audits after each intervention. These audit periods respectively spanned December 2024 to April 2025, January 2026 to May 2026, and June 2026 to July 2026. The project was registered locally with the clinical audit and quality improvement team and was exempted from formal IRB approval. All patient records were de-identified prior to analysis.

Interventions

Following the baseline audit, intervention 1 was implemented in April 2025 after user research and stakeholder engagement. This involved the transition from paper ward-round documentation to electronic free-text entries on iPortal, the electronic clinical record system already in use across the hospital. Implementation was supported by in-person guidance sessions for new resident doctors, verbal reminders, and visual guidance in the form of a poster highlighting the importance of complete documentation in the resident doctors’ office. No additional planned documentation intervention was introduced before Audit 2, which was undertaken from January to May 2026 after a period of routine use of electronic free-text documentation.

The second intervention was implemented in June 2026 following Audit 2. This was a user-informed structured electronic ward round proforma, shown in Figure 1. The proforma contained fixed fields for recording presenting complaint, date of admission, medical and surgical background and comorbidities, relevant investigations and imaging results, ReSPECT form status, and venous thromboembolism (VTE) risk assessment, alongside a free-text field for recording daily ward round updates. None of these fields was mandatory. During Audit 3, clinicians could use either the proforma or standard electronic free-text documentation; the proforma was therefore not enforced as the sole documentation method, allowing assessment of real-world uptake and contemporaneous performance.

**Figure 1:**
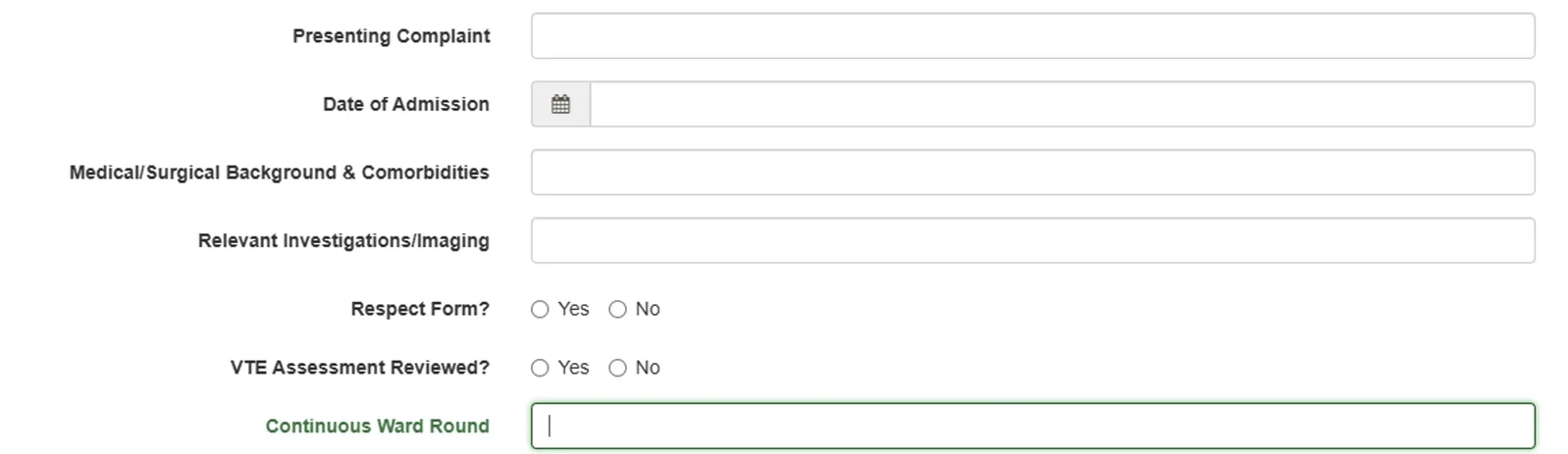
Screenshot of empty structured ward round proforma. VTE: venous thromboembolism.

Data collection and analysis

Audit standards were set based on adaptations of the RCSEd SHINE Surgical Ward Round Toolkit, developed collaboratively by the Patient Safety Committee of the Royal College of Surgeons of Edinburgh and the Royal Infirmary of Edinburgh [9]. The toolkit is freely available from the RCSEd website, and Tool 3 is explicitly provided in an editable format for adaptation to individual units. Each ward round entry was assessed for documentation of the following 11 criteria: history of presenting complaint, physical examination findings, most recent National Early Warning Score (NEWS), recent laboratory results, recent relevant radiology results, VTE risk assessment, antibiotic name where relevant, pain assessment and/or analgesia requirements, eating or nil-by-mouth (NBM) status, hydration status, and escalation and/or do not attempt cardiopulmonary resuscitation (DNACPR) status. For each ward round entry, each criterion was coded as documented, not documented, or not clinically applicable. Coding assessed whether the relevant information was present in the ward-round entry rather than the clinical accuracy or quality of the information recorded. “Not clinically applicable” was used when a criterion was not relevant to that entry; these observations were excluded from criterion-specific denominators and completeness calculations. Applicability was determined pragmatically by the reviewing doctors based on the clinical context of the entry, with uncertainty resolved by consensus.

The sampling unit was an individual inpatient urology ward-round entry. A pragmatic sample of entries was drawn across multiple ward rounds, dates, and patients to reduce reliance on a single clinical team or documenter. No entry was excluded solely because an individual criterion was not clinically applicable. Although repeat entries from the same patient were not excluded by design, no patient appeared more than once in the analysed sample. Clinician identifiers were not retained in the analysis dataset, so any within-documenter clustering could not be quantified. Data were collected by five resident doctors, and entries were assessed jointly, with disagreements on classification resolved by consensus to standardise data collection. Audit 1 (baseline) assessed a total of 32 handwritten paper note entries between December 2024 and April 2025. Audit 2 assessed 43 electronic free-text ward round entries after the first intervention between January 2026 and May 2026. Audit 3 was completed after the electronic proforma was launched, assessing 50 ward round entries, including both proforma and continued free-text notes, between June 2026 and July 2026. The audit periods are summarised in Table 1. No formal sample size calculation was performed because this project was undertaken as a quality improvement initiative, with the aim of obtaining a sample across each audit period while remaining feasible for manual review. As the study was not designed to test a prespecified effect size, a conventional power calculation was not considered appropriate. The third audit was intentionally larger to allow assessment of both proforma and continued free-text documentation following implementation of the intervention.

**Table 1:** Audit timeline and sequential interventions.

Stage	Time period	Documentation/Intervention	Entries
Audit 1: baseline	December 2024 - April 2025	Handwritten paper notes	32
Intervention 1	April 2025	Electronic free-text; staff education, verbal reminders, and visual guidance	n/a
Audit 2: re-audit	January - May 2026	Electronic free-text notes	43
Intervention 2	June 2026	Structured electronic ward-round proforma	n/a
Audit 3: post-proforma implementation	June - July 2026	Proforma or continued electronic free-text	50

Per-entry completeness was calculated as the primary outcome - this is the number of applicable criteria documented divided by the total number of applicable criteria for that entry. Hence, non-applicable criteria were excluded. Each applicable criterion contributed equally to the per-entry completeness score; no differential weighting was applied. Additionally, for each audit period, aggregate compliance was calculated as the proportion of all applicable documentation criteria that were coded as documented.

Statistical analysis

Per-entry completeness across the three audit periods was compared using the Kruskal-Wallis test. Where the overall test was significant, pairwise two-sided Mann-Whitney U tests were performed with Holm correction across the three comparisons. Within Audit 3, per-entry completeness for proforma and contemporaneous free-text entries were compared using a two-sided Mann-Whitney U test. Criterion-level documented versus not-documented counts were compared using two-sided Fisher's exact tests after exclusion of observations coded as not clinically applicable, with Holm correction across the 11 criterion-level comparisons. Statistical significance was defined as p < 0.05. Analyses were performed in Microsoft Excel (Microsoft Corporation, Redmond, WA) using the Real Statistics Resource Pack; tied ranks were handled using the software's standard procedures. No imputation was performed.

## Results

Per-entry completeness was higher in each successive audit period (Table 2 and Figure 2). Median per-entry completeness for paper documentation was 27.3% (IQR: 19.5%-40.0%), 50.0% (IQR: 40.0%-60.0%) for electronic free-text notes, and 60.0% (IQR: 54.5%-72.7%) during the post-proforma implementation period. These differences were statistically significant (Kruskal-Wallis H = 42.83, p < 0.001). Pairwise Mann-Whitney U tests showed significant differences between paper and electronic free-text (U = 266, p < 0.001), between electronic free-text and the post-proforma period (U = 672.5, p = 0.002), and between paper and the post-proforma period (U = 168.5, p < 0.001). All three pairwise comparisons remained statistically significant after Holm correction. Aggregate compliance increased from 29.2% (95/325) with paper notes to 48.3% (207/429) with electronic free-text notes, and 59.3% (293/494) during the post-proforma period.

**Table 2:** Per-entry documentation completeness across audit cycles. Data are presented as the median and interquartile range (IQR) of per-entry completeness percentages. Differences across the three audit periods were assessed using the Kruskal-Wallis test (H = 42.83, p < 0.001). Statistical significance was defined as p < 0.05.

Stage	n	Median (%)	IQR
Audit 1: baseline (paper)	32	27.3	19.5-40.0
Audit 2: electronic free-text	43	50.0	40.0-60.0
Audit 3: post-proforma implementation	50	60.0	54.5-72.7

**Figure 2:**
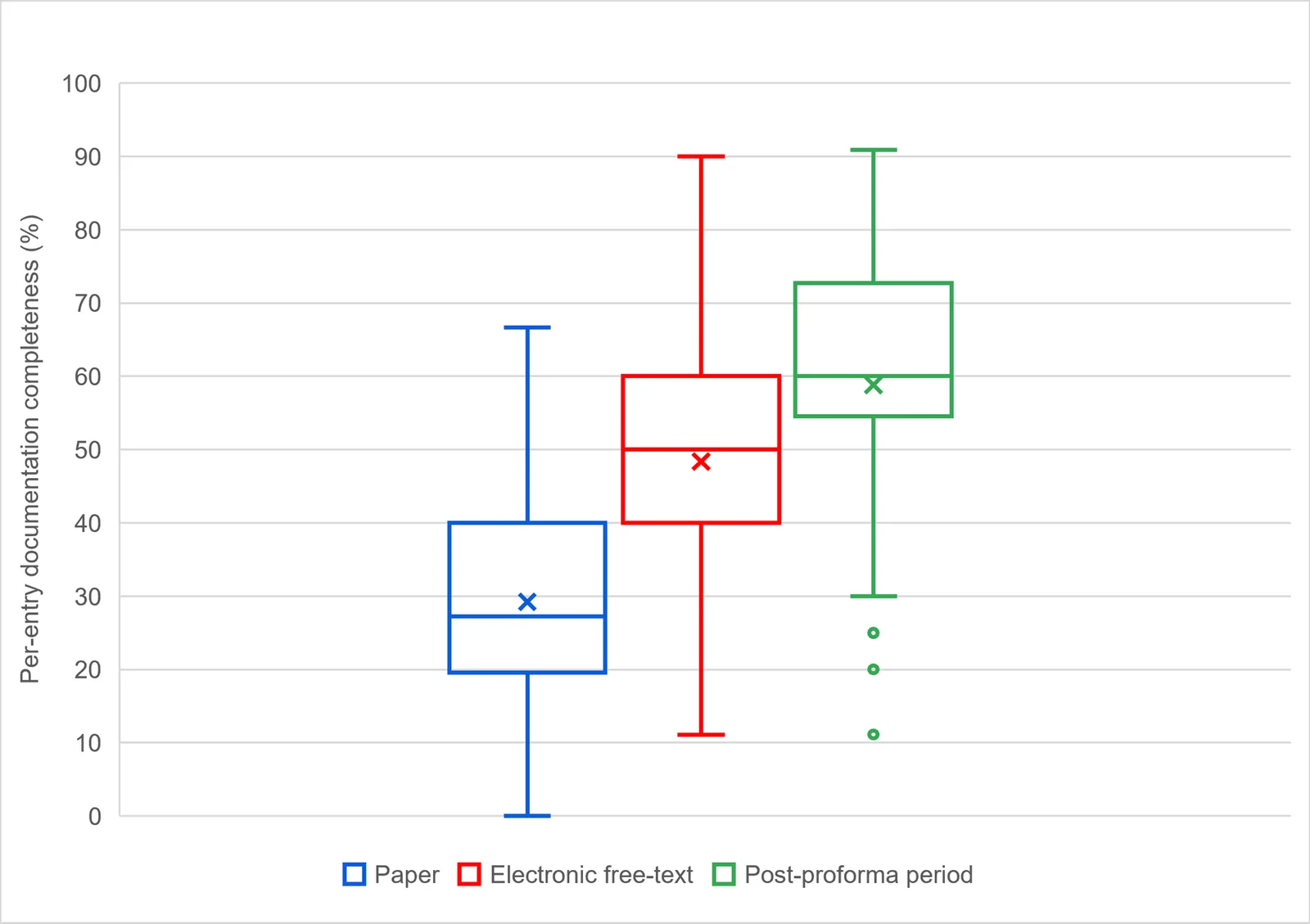
Distribution of per-entry documentation completeness across audit cycles. Box-and-whisker plots show per-entry completeness for paper documentation (n = 32), electronic free-text documentation (n = 43), and the post-proforma implementation period (n = 50). Boxes represent the interquartile range (IQR), with the horizontal line indicating the median. Whiskers extend to the most extreme non-outlying observations, and points beyond the whiskers are displayed as outliers. Mean values are shown as markers. Quartiles were calculated using the inclusive median method. Differences across the three audit periods were assessed using the Kruskal-Wallis test (H = 42.83, p < 0.001). Statistical significance was defined as p < 0.05.

Among the 50 ward round notes assessed in the post-proforma implementation period, the proforma was used in 52% (26/50) of entries. Proforma entries had a higher median per-entry completeness than contemporaneous free-text notes (63.6% (IQR: 56.0%-76.5%) vs. 55.1% (IQR: 35.6%-65.2%); Mann-Whitney U = 449, p = 0.008) as well as higher aggregate compliance rates (65.9% (174/264) vs. 51.7% (119/230)). We observed numerically higher compliance among proforma notes for nine of the 11 criteria (Table 3). The largest observed differences were in relevant radiology results (100.0% (20/20) vs. 53.3% (8/15)), physical examination findings (96.2% (25/26) vs. 79.2% (19/24)), and NEWS (92.3% (24/26) vs. 70.8% (17/24)); however, only relevant radiology results remained statistically significant after Holm correction of the 11 criterion-level Fisher’s exact tests (adjusted p = 0.011). Documentation of VTE risk assessment and escalation/DNACPR status remained poor regardless of method.

**Table 3:** Criterion-level documentation compliance: proforma versus contemporaneous free-text entries. Data are presented as n/N (%), where n is the number of entries in which the criterion was documented, and N is the number of entries for which the criterion was clinically applicable; entries coded as not applicable were excluded. Criterion-level comparisons were performed using two-sided Fisher's exact tests. P-values were adjusted for 11 comparisons using the Holm method. Statistical significance was defined as Holm-adjusted p < 0.05. NEWS: National Early Warning Score; VTE: venous thromboembolism; NBM: nil by mouth; DNACPR: do not attempt cardiopulmonary resuscitation.

Criterion	Proforma compliance	Free-text compliance	Fisher's exact p	Holm-adjusted p
History of presenting complaint	100.0% (26/26)	91.7% (22/24)	0.2253	1.000
Physical examination	96.2% (25/26)	79.2% (19/24)	0.0925	0.8325
NEWS	92.3% (24/26)	70.8% (17/24)	0.0693	0.6926
Recent laboratory tests	95.8% (23/24)	82.6% (19/23)	0.1882	1.000
Recent radiology	100.0% (20/20)	53.3% (8/15)	0.0010	0.0105
VTE assessment	26.9% (7/26)	20.8% (5/24)	0.7447	1.000
Antibiotic name	52.2% (12/23)	54.5% (6/11)	1.0000	1.000
Pain assessment/analgesia	42.3% (11/26)	20.8% (5/24)	0.1354	1.000
Eating/NBM	61.1% (11/18)	38.9% (7/18)	0.3175	1.000
Hydration	56.5% (13/23)	57.9% (11/19)	1.0000	1.000
Escalation/DNACPR	7.7% (2/26)	0.0% (0/24)	0.4906	1.000

## Discussion

Prior to any interventions, staff feedback and baseline audit results highlighted significant concerns regarding ward round documentation, particularly surrounding omissions of key information as well as practical concerns such as illegibility and inaccessible paper notes.

Across successive audit periods following these interventions, ward round documentation completeness was progressively higher, guided by compliance with criteria adapted from the RCSEd SHINE Surgical Ward Round Toolkit [9]. This is consistent with literature reporting improved documentation following structured ward-round interventions and is in line with national recommendations for ward rounds [4,5,11]. Possible explanations for the observed differences include legibility advantages in online documentation, user-dependent ease and speed of typing online notes, and easier access to prior ward round entries. A previous comparison between paper and electronic ward round documentation reported greater staff satisfaction with electronic notes [12]. While this was not formally assessed in this project, factors underlying improved staff experience with electronic documentation may also partly explain the differences observed in our setting.

Digitalisation alone did not, however, address poor documentation of VTE risk assessment and escalation status. This contrasts with previous proforma-based interventions that have reported improvements in documentation of VTE prophylaxis and resuscitation and/or escalation plans [13,14]. This highlights a need for targeted implementation strategies beyond digitalisation of documentation alone. Zarkali et al. demonstrated one method of supplementing a proforma with a structured handover intervention to target improvements in documentation of escalation plans, and a similar approach may be warranted in our setting [15].

Analysis of ward round notes in the post-proforma implementation period showed higher compliance with documentation standards in proforma entries, as demonstrated by higher median per-entry completeness. At criterion-level, proforma use was associated with higher compliance in nine of 11 criteria, with only radiology result documentation remaining statistically significant after correction for multiple comparisons. Reasons for this may include our modest sample sizes, several criteria already being highly compliant in the post-proforma period and thus producing a ceiling effect, as well as there being a dedicated field in the proforma for radiology results, which may have acted as a direct prompt to ensure documentation of this.

Notably, proforma uptake was suboptimal. Potential barriers to using the proforma, which were not formally measured in this project, may include unfamiliarity with its use, lack of awareness of its existence, perceived difficulty and/or increased documentation time, or variable endorsement from senior colleagues. This is similar to the findings of a previous implementation of a ward round template by Gilliland et al. in which documentation improved, while noting challenges in template utilisation itself [6]. Implementation factors may therefore be as important as the design of the proforma itself. Pitcher et al. found that introduction of a structured ward round form alone did not initially improve documentation, with improvement occurring after specific education in its use [7], while Krishnamohan et al. highlighted senior clinician and nursing engagement as important for sustained checklist use [8].

There remain several limitations in this quality improvement project. This was a single-centre project with modest and unequal sample sizes. The sequential before-and-after design and different audit periods mean that changes in staffing, workload, case mix, documentation attitudes, or other contextual factors could have contributed to the observed differences, and causality therefore cannot be established. Patient-level characteristics were not systematically collected for case-mix adjustment. Doctors choosing to use the proforma may have been more conscientious documenters, introducing selection bias. Although no patient appeared more than once in the analysed sample, clinician identifiers were not retained, so possible within-documenter clustering could not be assessed. Entries were assessed jointly, and disagreements were resolved by consensus, but independent duplicate ratings were not collected; formal inter-rater reliability therefore could not be calculated. In addition, detailed operational definitions for clinical applicability were not prospectively standardised, which may have allowed some variability in classification to persist despite consensus review. The per-entry completeness score was derived from adapted SHINE criteria but was not formally validated as a composite measure; applicable criteria were weighted equally, and the score assessed whether information was documented rather than its accuracy, clarity, or clinical usefulness. Implementation and balancing outcomes, including documentation time, usability, workflow burden, and sustainability, were not formally measured. Furthermore, this audit measured documentation processes as opposed to direct patient outcomes such as adverse events; higher documentation completeness therefore cannot be assumed to represent improved clinical outcomes or patient safety.

Future work should address the above limitations, including prospective standardisation of applicability definitions and assessment of implementation and balancing measures such as documentation time, usability, and sustainability. In addition, subsequent quality improvement work should consider how to improve uptake of the proforma, as well as targeted interventions for increasing documentation of VTE risk assessment and escalation status, which remained poorly documented. This process should be supported by further stakeholder engagement to identify workflow barriers, while ensuring that efforts to improve documentation completeness do not increase administrative burden or impair usability.

## Conclusions

This quality improvement project supported the transition of ward round documentation from handwritten paper notes to electronic free-text documentation, followed by the implementation of a structured online proforma. Across these sequential audit periods, documentation completeness was significantly higher in later audits. Proforma use was associated with greater completeness than contemporaneous free-text notes. These findings relate to documentation completeness and do not establish that the interventions caused the observed differences or that clinical outcomes or patient safety improved. The observed changes highlight the potential for clinical audit cycles, stakeholder engagement, and iterative implementation to support changes in documentation practice.

VTE risk assessment and escalation/DNACPR status remained poorly documented despite these interventions, and there was incomplete uptake of the proforma. Future quality improvement work should focus on increasing adoption alongside targeted proforma refinement.
